# A Case Report of Bilateral Adrenal Sarcomatoid Carcinoma

**DOI:** 10.1155/2016/3768258

**Published:** 2016-12-20

**Authors:** Ozlem Turhan Iyidir, Ethem Turgay Cerit, Çiğdem Özkan, Eroğlu Altınova, Ali Rıza Çimen, Sinan Sözen, Mustafa Kerem, Müjde Aktürk, Leyla Memiş, Baloş Törüner, Nuri Çakır, Metin Arslan

**Affiliations:** ^1^Department of Endocrinology and Metabolism, Gazi University Faculty of Medicine, Ankara, Turkey; ^2^Department of Urology, Gazi University Faculty of Medicine, Ankara, Turkey; ^3^Department of Surgery, Gazi University Faculty of Medicine, Ankara, Turkey; ^4^Department of Pathology, Gazi University Faculty of Medicine, Ankara, Turkey

## Abstract

Adrenocortical carcinoma (ACC) is a rare and aggressive malignancy. Sarcomatoid adrenal carcinoma is even more aggressive type of ACC. Bilateral malignant adrenal tumors are extremely rare except for those that represent metastasis from an extra-adrenal organ. Here we report a 53-year-old woman who presented with abdominal pain and weight loss. Abdominal computed tomography revealed bilateral adrenal masses and a mass in her liver. Surgical specimens showed pleomorphic tumor cells with epithelial and spindle cell morphology and immunohistochemical staining was compatible with sarcomatoid carcinoma. Sarcomatoid adrenal carcinoma should be kept in mind during the management of bilateral adrenal masses.

## 1. Introduction

Adrenal masses are found incidentally during radiological examinations performed for indications other than adrenal diseases. Incidental adrenal masses are reported as 8.7% and bilateral masses account for 10–15% of the cases [1, 2]. The most frequent causes for bilateral adrenal masses are metastatic disease, congenital adrenal hyperplasia, cortical adenomas, lymphoma, infections (e.g., tuberculosis, fungal), hemorrhage, corticotropin (ACTH) dependent Cushing's disease, pheochromocytoma, amyloidosis, infiltrative disease of the adrenal glands, and bilateral macronodular adrenal hyperplasia. Adrenocortical carcinoma (ACC) is bilateral in 10% cases and should also be considered for differential diagnosis of bilateral adrenal masses [3]. Sarcomatoid carcinoma is a biphasic tumor with sarcoma like or sarcomatoid component and has a poor prognosis with rapid fatal behavior. Adrenal sarcomatoid carcinoma is a very rare malignant tumor and to date there are only 19 cases reported in the literature and to the best of our knowledge there has been only one case with bilateral disease [4].

Here, we report a patient who was admitted with bilateral adrenal masses and diagnosed as adrenal sarcomatoid carcinoma.

## 2. Case Report

**Figure 1:**
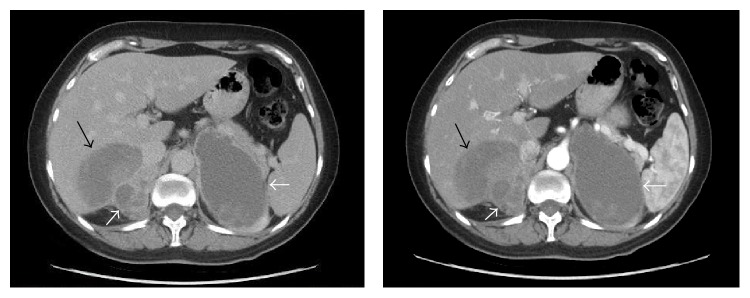
Computed tomography (CT) of abdomen; cystic masses replacing both adrenal glands and infiltrating liver can be seen (arrows).

53-year-old woman was admitted to the hospital with a 1-month history of abdominal and flank pain and 10 kilograms of weight loss in three months. She had no remarkable medical history except a 10 pack-year history of smoking. On her physical examination, blood pressure was 120/70 mmHg and her pulse was 72/min. There were no signs and symptoms of adrenal hormonal hypersecretion. Abdominal computed tomography (CT) scan showed 10.5 × 6.5 × 9.5 cm cystic mass with irregular borders located in left adrenal and 6.5 × 3.5 × 4.5 cm lobulated cystic mass with irregular borders and multiple septations in the right adrenal gland. There was also an 8.3 × 6.5 cm cystic mass with irregular borders in liver segment six and the lower margin of this mass could not be clearly differentiated from the borders of the mass described in right adrenal on CT scan (Figure 1).

**Figure 2:**
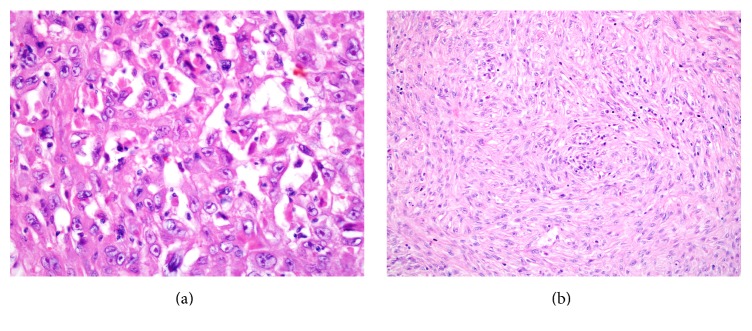
Microscopic findings of the resected tumor. (a) Epithelioid cells display vesicular nucleus and mitosis (H&E ×40), (b) fascicles of spindle cells (H&E ×20).

Serum cortisol, serum androgens, urine catecholamines and their metabolites, and the aldosterone to renin ratio were normal. Her liver function tests including ALT and AST were four times higher than normal and renal function tests were normal. Mammography, upper gastrointestinal system endoscopy, and thorax CT were performed to exclude metastasis to adrenals, which gave normal results. Bilateral adrenalectomy, simple nephrectomy, distal pancreatectomy, splenectomy, right hepatectomy, and cholecystectomy were performed. The surgery was completed uneventfully. On gross examination specimen removed from right adrenal was brown and nodular measuring 9 × 6.5 × 4.0 cm and weighed 80 g. Its cut surface showed that the tumor was variegated with whitish-grey firm solid areas and extensive necrosis. The specimen removed from left adrenal measured 8.5 × 6.0 × 3.5 cm and was composed of cystic mass. On microscopic examination the tumor of right and left adrenal showed pleomorphic tumor cells with epithelial and spindle cell morphology with evidence of foci of necrosis. Giant cell formation and high mitotic rates were observed in the sarcomatous component (Figure 2). Upon histopathological examination, immunohistochemical staining demonstrated diffuse positivity for vimentin and pan-cytokeratin and focal positivity for S-100 protein, epithelial membrane antigen (EMA), desmin, chromogranin, and CD 31; negativity for synaptophysin, calretinin, melan-a, *α*-inhibin, HBM-45 (human melanoma black 45), keratin 5/6, HCC, ACTH, SMA (Smooth Muscle Antigen), Myoglobin, Myogenin, and Calponin. The final diagnosis was consistent with primary adrenocortical sarcomatoid carcinoma of both right and left adrenal glands.

After the operation, pancreatic fistula developed during treatment in the intensive care unit and patient's general condition deteriorated rapidly. One month after surgery the patient died because of multiorgan failure.

## 3. Discussion

ACC is a rare and highly aggressive malignancy accounting for an estimated 0.02% of all cancers [5]. Adrenal tumors are mostly sporadic and unilateral but some of them are bilateral and associated with Li-Fraumeni Syndrome, Type-1 Multiple Endocrine Neoplasia, Beckwith-Wiedemann Syndrome, and Carney complex [6]. When bilateral adrenal masses are incidentally discovered, a workup for another primary malignancy foci should be done. Regarding bilaterality of the adrenal masses of the present case, we initially did a diagnostic workup to exclude metastasis from extra-adrenal organs. The primary cancers that most often spread to the adrenals are those of the lung, gastrointestinal tract, breast, kidney, and liver [7, 8]. In this context our patient's mammography, colonoscopy, upper gastrointestinal system endoscopy, and thorax CT were normal. Since we could not show any extra-adrenal malignant foci, adrenocortical carcinoma was our initial diagnosis. According to the recent guidelines adrenal biopsy is not an option for the patient's with an adrenal mass which is likely to be adrenocortical carcinoma because of the risk of tumor dissemination [9]. The accurate diagnosis could be made after surgical exploration. Histopathological examination of the adrenalectomy specimens revealed bilateral adrenal carcinoma with sarcomatous component.

Adrenal sarcomatoid carcinoma is a rare morphological variant of ACC and features of carcinoma are combined with those of sarcoma [10]. Three of the reported cases were metastatic to liver [11], inferior vena cava [12], and lung [13]. One patient had bilateral adrenal sarcomatoid carcinoma [4]. Our patient had bilateral adrenal sarcomatoid carcinoma and liver metastasis at the time of diagnosis. We could easily excluded metastasis from a primary neoplasm, but metastasis from one adrenal gland to another could not be completely ruled out since we did not have any previous imaging studies for adrenal glands before admission.

Based on the data collected from previous cases, primary adrenal sarcomatoid carcinoma is mainly seen in middle-aged patients and male : female ratio is 1 : 1. Twelve of the cases died within 2 days to 30 months after the diagnosis [13]. Our case was a middle-aged woman and died 2 months after diagnosis.

According to previous cases sarcomatoid carcinomas originating from adrenal cortex are mostly composed of malignant spindle cells without any identifiable heterologous differentiation [14]. Our case's adrenal histopathology revealed prominent population of malignant spindle cells.

Definitive diagnosis of sarcomatoid carcinoma can be made easily if there is a distinctive differentiation into adrenocortical cells. However the diagnosis is much more complicated especially when the differentiation is indistinctive and when tumors present bilaterally and immunostaining should be employed [4]. Positivity with calretinin and/or *α*-inhibin is known to be of highly diagnostic value for adrenocortical carcinoma [15], although all of these immunohistochemical markers were negative in the present case. Since positive frequency for these markers varies depending on the differentiation of the tumor, this could be explained with poor differentiation of the tumor of our patient. Our patient's imaging studies did not reveal any extra-adrenal primary foci and immunostaining was negative for the possible metastatic tumors such as renal cell carcinoma, lung cancer, lymphoma, and breast cancer.

Patients with an adrenal sarcomatoid tumor have very poor prognosis probably because of the stage of disease at the time of diagnosis. After removal of the tumor, adjuvant chemotherapy is suggested. Our patient could not receive any chemotherapy because of her poor performance status after surgery and she died because of multiorgan failure just one month after surgery. Adrenal sarcomatoid carcinoma cases with adrenal hormonal production have been reported previously; however there was no hormonal overproduction in our case [16].

She also did not have adrenal insufficiency despite bilateral adrenal masses.

To the best of our knowledge, our case is the second case of bilateral adrenal sarcomatoid carcinoma reported in the literature. In conclusion possible diagnosis of adrenal sarcomatoid carcinoma should be kept in mind during the management of bilateral adrenal masses. There is need for more data from similar cases.
